# Supplementary material for: Causal Relationship between Aging and Anorexia Nervosa: A White-Matter-Microstructure-Mediated Mendelian Randomization Analysis
Source: Biomedicines. 2024 Aug 16;12(8):1874. doi: 10.3390/biomedicines12081874 (PMC11351342; doi:10.3390/biomedicines12081874)
Supplement: Supplementary file 1 [file biomedicines-12-01874-s001.zip › Supplementary Figure/Figure S4/Singe SNP plot of MR analysis for TL to ubm-b-1719.pdf]

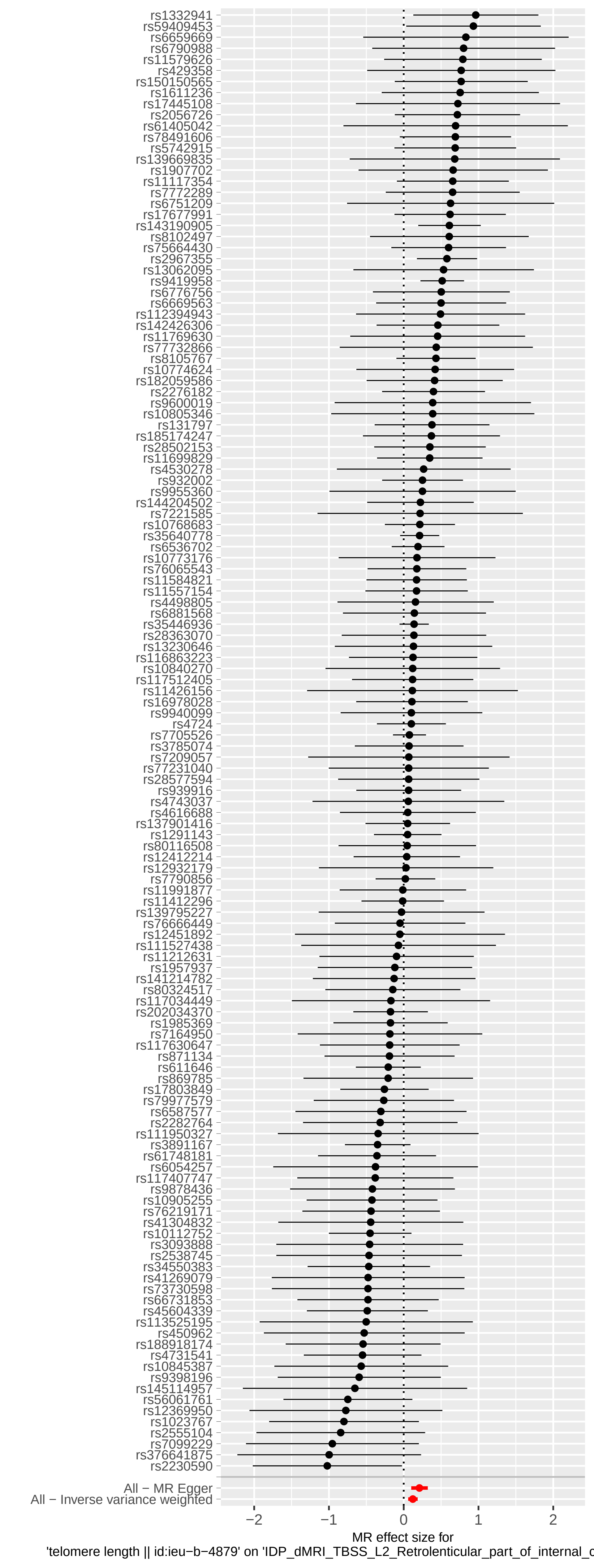
rs1332941
rs59409453
rs6659669
rs6790988
rs11579626
rs429358
rs150150565
rs1611236
rs17445108
rs2056726
rs61405042
rs78491606
rs5742915
rs139669835
rs1907702
rs11117354
rs7772289
rs6751209
rs17677991
rs143190905
rs8102497
rs75664430
rs2967355
rs13062095
rs9419958
rs6776756
rs6669563
rs112394943
rs142426306
rs11769630
rs77732866
rs8105767
rs10774624
rs182059586
rs2276182
rs9600019
rs10805346
rs131797
rs185174247
rs28502153
rs11699829
rs4530278
rs932002
rs9955360
rs144204502
rs7221585
rs10768683
rs35640778
rs6536702
rs10773176
rs76065543
rs11584821
rs11557154
rs4498805
rs6881568
rs35446936
rs28363070
rs13230646
rs116863223
rs10840270
rs117512405
rs11426156
rs16978028
rs9940099
rs4724
rs7705526
rs3785074
rs7209057
rs77231040
rs28577594
rs939916
rs4743037
rs4616688
rs137901416
rs1291143
rs80116508
rs12412214
rs12932179
rs7790856
rs11991877
rs11412296
rs139795227
rs76666449
rs12451892
rs111527438
rs11212631
rs1957937
rs141214782
rs80324517
rs117034449
rs202034370
rs1985369
rs7164950
rs117630647
rs871134
rs611646
rs869785
rs17803849
rs79977579
rs6587577
rs2282764
rs111950327
rs3891167
rs61748181
rs6054257
rs117407747
rs9878436
rs10905255
rs76219171
rs41304832
rs10112752
rs3093888
rs2538745
rs34550383
rs41269079
rs73730598
rs66731853
rs45604339
rs113525195
rs450962
rs188918174
rs4731541
rs10845387
rs9398196
rs145114957
rs56061761
rs12369950
rs1023767
rs2555104
rs7099229
rs376641875
rs2230590
All − MR Egger
All − Inverse variance weighted
−2
−1
0
1
2
MR effect size for
'telomere length || id:ieu−b−4879' on 'IDP_dMRI_TBSS_L2_Retrolenticular_part_of_internal_c
